# GDF-11 downregulates placental human chorionic gonadotropin expression by activating SMAD2/3 signaling

**DOI:** 10.1186/s12964-023-01201-5

**Published:** 2023-07-21

**Authors:** Ze Wu, Lingling Zhang, Yuanyuan Jia, Beibei Bi, Lanlan Fang, Jung-Chien Cheng

**Affiliations:** Center for Reproductive Medicine, Henan Key Laboratory of Reproduction and Genetics, The First Affiliated Hospital of Zhengzhou University, 40, Daxue Road, Zhengzhou, 450052 Henan China

**Keywords:** GDF-11, Trophoblast cells, CGB, SMAD

## Abstract

**Background:**

The production of human chorionic gonadotropin (hCG) by the placental trophoblast cells is essential for maintaining a normal pregnancy. Aberrant hCG levels are associated with reproductive disorders. The protein of hCG is a dimer consisting of an α subunit and a β subunit. The β subunit is encoded by the CGB gene and is unique to hCG. Growth differentiation factor-11 (GDF-11), a member of the transforming growth factor-β (TGF-β) superfamily, is expressed in the human placenta and can stimulate trophoblast cell invasion. However, whether the expression of CGB and the production of hCG are regulated by GDF-11 remains undetermined.

**Methods:**

Two human choriocarcinoma cell lines, BeWo and JEG-3, and primary cultures of human cytotrophoblast (CTB) cells were used as experimental models. The effects of GDF-11 on CGB expression and hCG production, as well as the underlying mechanisms, were explored by a series of in vitro experiments.

**Results:**

Our results show that treatment of GDF-11 downregulates the expression of CGB and the production of hCG in both BeWo and JEG-3 cells as well as in primary CTB cells. Using a pharmacological inhibitor and siRNA-mediated approach, we reveal that both ALK4 and ALK5 are required for the GDF-11-induced downregulation of CGB expression. In addition, treatment of GDF-11 activates SMAD2/3 but not SMAD1/5/8 signaling pathways. Moreover, both SMAD2 and SMAD3 are involved in the GDF-11-downregulated CGB expression. ELISA results show that the GDF-11-suppressed hCG production requires the ALK4/5-mediated activation of SMAD2/3 signaling pathways.

**Conclusions:**

This study not only discovers the biological function of GDF-11 in the human placenta but also provides important insights into the regulation of the expression of hCG.

Video Abstract

**Supplementary Information:**

The online version contains supplementary material available at 10.1186/s12964-023-01201-5.

## Introduction

The mammalian placenta is a unique organ that is essential for establishing normal pregnancy. Chorionic villi represent the structural and functional unit of the human placenta and are composed of two cell layers. The outer layer is comprised of large multinucleated syncytiotrophoblast (STB) cells that are formed by the fusion of underlying cytotrophoblast (CTB) cells [1]. The syncytiotrophoblast layer is the placental barrier between maternal and fetal blood that allows the exchange of nutrients and gases and also acts as critical endocrine tissue. The syncytiotrophoblast cells can secret several steroid and peptide hormones. Among them, human chorionic gonadotropin (hCG) is responsible for maintaining the production of progesterone by binding to the luteinizing hormone (LH) receptor on the corpus luteum in the first trimester of pregnancy [2, 3]. In addition, hCG also regulates different physiological functions of extravillous trophoblasts, myometrial smooth muscle cells, endometrium cells, and vascular endothelial cells during pregnancy [4].

The hCG is a dimer consisting of an α subunit and a β subunit. The β subunit is encoded by the CGB gene and is unique to hCG. The α subunit is identical to that of LH, follicle-stimulating hormone, and thyroid-stimulating hormone. Dysregulation of hCG production has been associated with various pregnancy-related disorders, such as miscarriages [5] and preeclampsia [6]. Therefore, the synthesis and secretion of hCG need to be finely regulated. The human choriocarcinoma cell lines, BeWo and JEG-3, can secret hCG and are used as cell models for studying the regulation of trophoblast hCG synthesis and production [7]. Using BeWo cells, previous studies have revealed that several transcription factors and histone modifications are involved in CGB expression [8–11]. The human leukocyte antigen G promotes CGB expression and hCG production in both BeWo and JEG-3 cells by activating the ERK1/2 signaling [12]. Our previous study shows that treatment with amphiregulin (AREG), an epidermal growth factor receptor (EGFR) ligand, stimulates CGB expression and hCG production in BeWo cells by inducing ERK1/2-mediated expression of the inhibitor of DNA-binding protein-3 [13].

Growth differentiation factor-11 (GDF-11), also known as bone morphogenetic protein-11 (BMP-11), belongs to the transforming growth factor-β (TGF-β) superfamily and is expressed during embryogenesis [14, 15]. In humans, GDF-11 is expressed in nearly all major tissues and organs, with the highest levels of that in the spleen, kidney, and brain [16–18]. However, to date, the physiological function of GDF-11 in female reproductive function is limited. We have reported that GDF-11 is expressed in the human ovary and can downregulate the expression of the steroidogenic acute regulatory protein in ovarian granulosa cells [19]. Using immunohistochemical analysis, the expression of GDF-11 protein is detected in the human placenta [20]. Our recent study demonstrates that GDF-11 stimulates human extravillous trophoblast cell invasion by upregulating the expression of matrix metalloproteinase-2 [21]. However, whether GDF-11 affects CGB expression and hCG production remains undetermined. Therefore, the present study was designed to explore the effect and related underlying molecular mechanisms of GDF-11 on CGB expression and hCG production in human trophoblast cells.

## Materials and methods

### Antibodies and reagents

All antibodies used in this study are summarized in Table 1. The recombinant human GDF-11 and BMP-4 were obtained from R&D systems. The SB431542 was obtained from Sigma. GDF-11 and BMP-4 were solubilized in phosphate-buffered saline (PBS). SB431542 was dissolved in dimethyl sulfoxide (DMSO).

**Table 1:** The information of antibodies

Name of antibody	Manufacturer and catalog #	Molecular weight	Dilution used
CGB	Proteintech (11615–1-AP)	18 kDa	3000x
Phospho-SMAD1/5/8	Cell Signaling Technology (13820)	60 kDa	1000x
Phospho-SMAD2	Cell Signaling Technology (3108)	60 kDa	1000x
Phospho-SMAD3	Cell Signaling Technology (9520)	52 kDa	1000x
SMAD1	Cell Signaling Technology (6944)	60 kDa	1000x
SMAD2	Cell Signaling Technology (3103)	60 kDa	1000x
SMAD3	Cell Signaling Technology (9523)	52 kDa	1000x
SMAD4	Cell Signaling Technology (38454)	70 kDa	1000x
α-Tubulin	Santa Cruz (sc-23948)	55 kDa	5000x

### Cell culture of human choriocarcinomas cell lines

The human choriocarcinoma cell lines, BeWo and JEG-3, were obtained from American Type Culture Collection through an official distributor in China (Beijing Zhongyuan Limited). Cells were cultured in a humidified atmosphere containing 5% CO_2_ and 95% air at 37 °C in Dulbecco’s Modified Eagle Medium/nutrient mixture F-12 Ham medium (DMEM/F-12; Gibco) supplemented with 10% FBS (HyClone), 100 U/mL of penicillin, and 100 μg/mL of streptomycin sulfate (Boster).

### Primary cytotrophoblast (CTB) cell isolation and culture

The study received institutional approval and was carried out in accordance with the guidelines from the Zhengzhou University Research Ethics Board. Primary human CTB cells were isolated from first-trimester placentas as previously described [22]. Briefly, chorionic villi were finely minced and digested for 1 h at 37°C with an enzyme cocktail containing 1 mg/mL type IV protease, 0.5 mg/mL type IV collagenase, and 50 µg/mL DNAse I (Sigma). An equal volume of DMEM/F-12 culture media containing 10% FBS was added to stop the enzyme activity. Tissue digests were then filtered through a 40 µM sieve (BD Biosciences), centrifuged at 1200 rpm for 3 min, and seeded in a culture dish. After 24 h culture, attached viable cells were collected by trypsinization, and CTB cells were isolated using the EasySep™ Human EpCAM Positive Selection Kit (STEMCELL Technologies) [23]. Cells were cultured in a humidified atmosphere containing 5% CO_2_ and 95% air at 37°C in DMEM/F-12 supplemented with 10% FBS, 100 U/mL of penicillin, and 100 μg/mL of streptomycin sulfate.

### Reverse transcription quantitative real-time PCR (RT-qPCR)

Total RNA was extracted with TRIzol (Invitrogen) according to the manufacturer’s instructions. RNA (1 μg) was reverse-transcribed into first-strand cDNA with the iScript Reverse Transcription Kit (Bio-Rad Laboratories). Each 20 μL qPCR reaction contained 1X SYBR Green PCR Master Mix (Applied Biosystems), 60 ng of cDNA, and 250 nM of each specific primer. The primers used were CGB, 5'-GTG TGC ATC ACC GTC AAC AC-3' (sense) and 5'-GGT AGT TGC ACA CCA CCT GA-3' (antisense); ALK4, 5'-TCT CTC CAC CTC AGG GTC TG-3' (sense) and 5'-GCC ATA CTT CCC CAA ACC GA-3' (antisense); ALK5, 5'-GTT AAG GCC AAA TAT CCC AAA CA-3' (sense) and 5'-ATA ATT TTA GCC ATT ACT CTC AAG G-3' (antisense); SMAD2, 5'-CCG AAA TGC CAC GGT AGA AA-3' (sense) and 5'-GGG CTC TGC ACA AAG ATT GC-3' (antisense); SMAD3, 5'-CCC CAG CAC ATA ATA ACT TGG-3' (sense) and 5'-AGG AGA TGG AGC ACC AGA AG-3' (antisense); and GAPDH, 5'-GAG TCA ACG GAT TTG GTC GT-3' (sense) and 5'-GAC AAG CTT CCC GTT CTC AG-3' (antisense). RT-qPCR was performed using an Applied Biosystems QuantStudio 12 K Flex Real-Time PCR system equipped with a 96-well optical reaction plate. The specificity of each assay was validated by melting curve analysis and by agarose gel electrophoresis of the PCR products. All of the RT-qPCR experiments were run in triplicate, and a mean value was used to determine the mRNA levels. RNase-free water and mRNA without RT were used as negative controls. Relative quantification of the mRNA levels was performed using the comparative Ct method with GAPDH as the reference gene and using the formula 2^–∆∆Ct^.

### Western blot

Cells were lysed in cell lysis buffer (Cell Signaling Technology) supplemented with a protease inhibitor cocktail (Sigma). The protein concentration was analyzed by the BCA protein assay kit (Pierce, Thermo Scientific). Equal amounts of protein were separated by SDS polyacrylamide gel electrophoresis and transferred onto PVDF membranes. After 1 h blocking with 5% non-fat dry milk in Tris-buffered saline (TBS), the membranes were incubated overnight at 4 °C with primary antibodies diluted in 5% non-fat milk/TBS. Following primary antibody incubation, the membranes were incubated with appropriate HRP-conjugated secondary antibodies. Immunoreactive bands were detected using an enhanced chemiluminescent substrate (Bio-Rad Laboratories) and imaged with a ChemiDoc MP Imager (Bio-Rad Laboratories). Band intensities were quantified using the Scion Image software.

### Small interfering RNA (siRNA) transfection

To knock down endogenous ALK4 (#L-004925–00-0010), ALK5 (#L-003929–00-0010), SMAD4 (#L-003902–00-0010), SMAD2 (#L-003561–00-0010), or SMAD3 (#L-020067–00-0010), cells were transfected with 50 nM (for BeWo and JEG-3) or 100 nM (for primary CTB) ON-TARGETplus SMARTpool siRNA targeting specific gene (Dharmacon) using Lipofectamine RNAiMAX (Invitrogen). The ON-TARGETplus Non-targeting Control Pool siRNA (#D-001810–10-50) (Dharmacon) was used as the transfection control. Cells were transfected with control or specific siRNA for 48 h. The efficiency of siRNA-mediated knockdown was determined by RT-qPCR or western blot.

### Measurement of hCG

hCG levels in culture media were measured using an hCG ELISA kit according to the manufacturer's instructions (Elabscience, #E-EL-H0175). Both inter-assay and intra-assay CV for hCG ELISA were < 10%. The analytical sensitivity of hCG ELISA was 4.69 mIU/mL. The hCG level in each culture medium was presented as an exact value and normalizing it to the total protein amount from the corresponding cell lysate (mIU/mL/µg protein).

### Statistical analysis

The results are presented as the mean ± SEM of at least three independent experiments. All statistical analyses were analyzed by the PRISM software. Multiple comparisons were analyzed using a one-way ANOVA followed by Tukey’s multiple comparison test. A significant difference was defined as *p* < 0.05.

## Results

### GDF-11 downregulates CGB expression in BeWo and JEG-3 cells

**Fig. 1:**
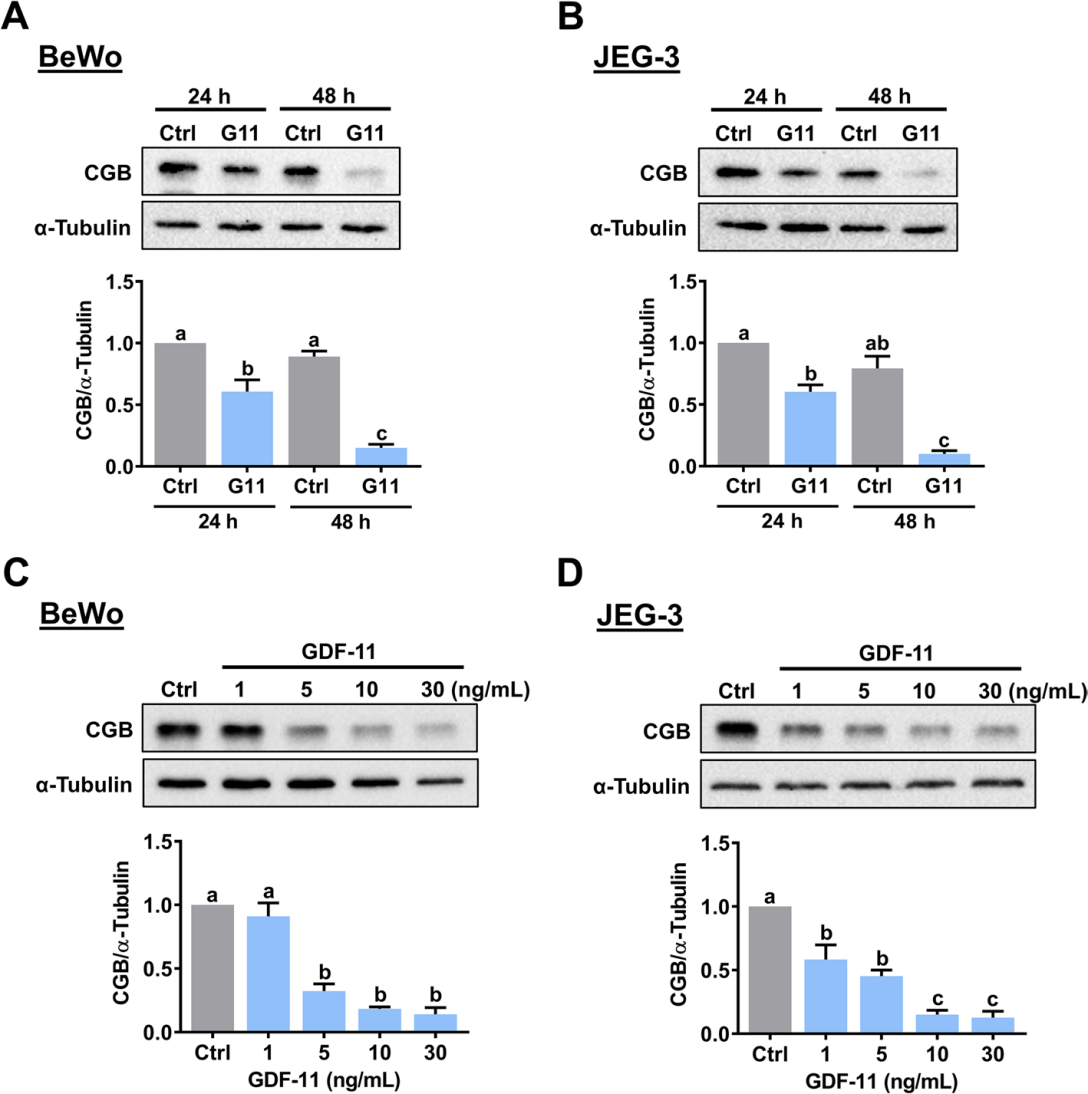
GDF-11 downregulates CGB expression in BeWo and JEG-3 cells. **A** and **B** BeWo (**A**) and JEG-3 (**B**) cells were treated with 30 ng/mL GDF-11 (G11) for 24 h or every 24 h for 48 h. The protein levels of CGB were examined by western blot. **C** and **D** BeWo (**C**) and JEG-3 (**D**) cells were treated with 1, 5, 10, and 30 ng/mL GDF-11 every 24 h for 48 h. The protein levels of CGB were examined by western blot. The results are expressed as the mean ± SEM of at least three independent experiments. The values without a common letter are significantly different (*p* < 0.05)

It has been shown that in women of reproductive age, the serum level of GDF-11 can reach 40 ng/mL [24]. To examine the effect of GDF-11 on the CGB expression, BeWo cells were treated with 30 ng/mL human recombinant GDF-11 for 24 and 48 h. Western blot analysis showed that 24 h of GDF-11 treatment significantly downregulated the protein levels of CGB in BeWo cells. A more profound suppressive effect of GDF-11 on CGB protein levels was observed after 48 h of GDF-11 treatment (Fig. 1A). Similarly, the inhibitory effect of GDF-11 on CGB protein levels was detected in another human choriocarcinoma cell line, JEG-3 (Fig. 1B). Since 30 ng/mL GDF-11 treatment resulted in a significant suppressive effect on CGB expression, concentration-dependent experiments were performed to examine the effects of lower concentrations of GDF-11 on CGB expression. As shown in Fig. 1C, while treatment of 1 ng/mL GDF-11 had no significant effect, CGB protein levels were significantly downregulated by exposure to 5, 10, or 30 ng/mL GDF-11. In JEG-3 cells, similarly, treatment with GDF-11 downregulated CGB protein levels in a concentration-dependent manner (Fig. 1D). Therefore, 10 ng/mL GDF-11 was used in the following experiments.

### GDF-11 downregulates CGB expression through ALK4 and ALK5

**Fig. 2:**
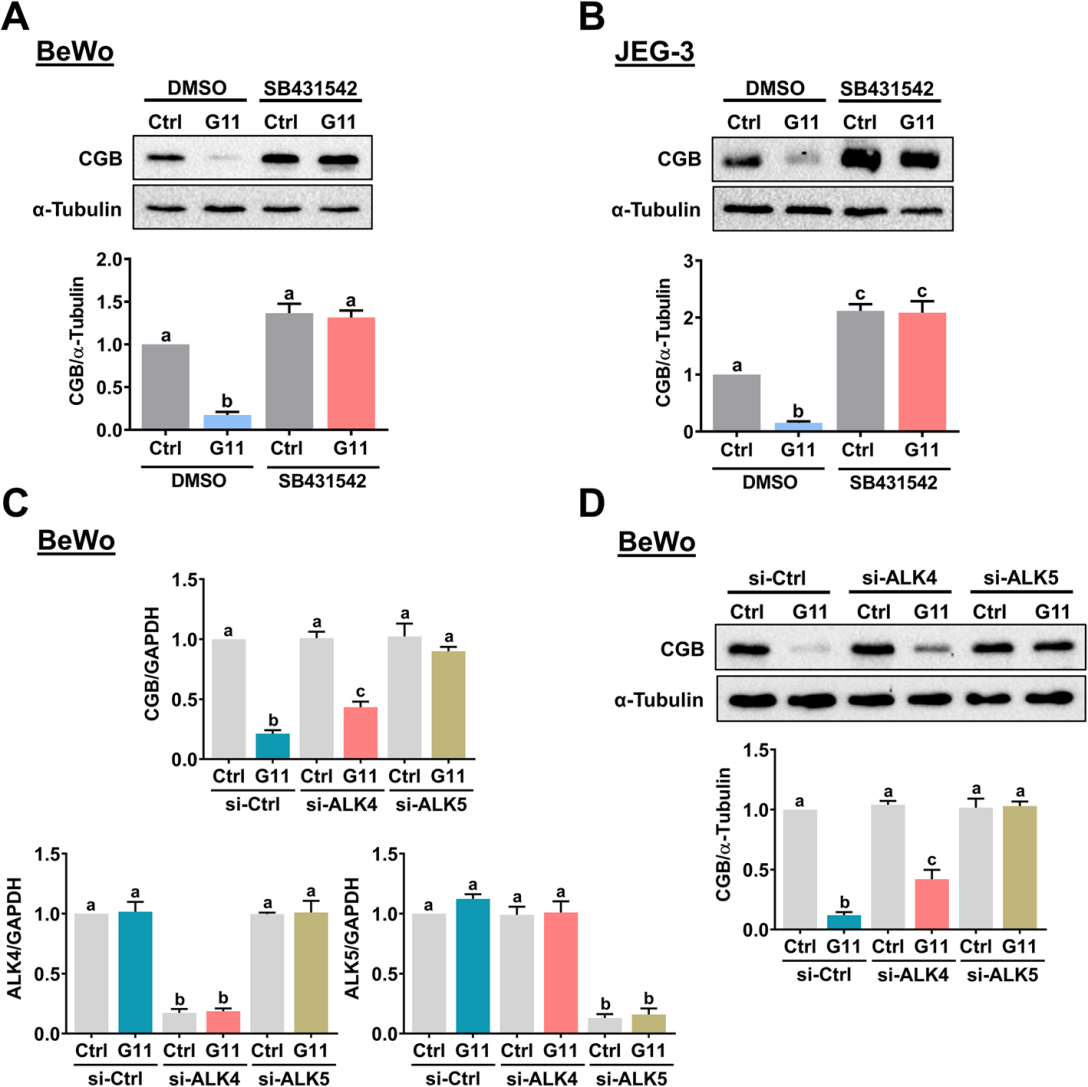
ALK4 and ALK5 are involved in the GDF-11-induced downregulation of CGB expression. **A** and **B** BeWo (**A**) and JEG-3 (**B**) cells were pretreated with vehicle control (DMSO) or 10 µM SB431542 for 1 h, and then treated with 10 ng/mL GDF-11 (G11) every 24 h for 48 h. The protein levels of CGB were examined by western blot. **C** and **D** BeWo cells were transfected with 50 nM control siRNA (si-Ctrl), ALK4 siRNA (si-ALK4), or ALK5 siRNA (si-ALK5) for 48 h, and then treated with 10 ng/mL GDF-11 (G11) every 24 h for 48 h. The mRNA (**C**) levels of ALK4, ALK5, and CGB were examined by RT-qPCR. The protein (**D**) levels of CGB were examined by western blot. The results are expressed as the mean ± SEM of at least three independent experiments. The values without a common letter are significantly different (*p* < 0.05)

GDF-11 exhibits its biological function by binding to two activin type-II receptors, ActRIIA and ActRIIB, and three type-I receptors, activin receptor-like kinase 4 (ALK4), ALK5, and ALK7 [25]. In a cell-type-dependent manner, GDF-11 regulates cellular function by using different ALKs. As the expression levels of ALK7 are very low in the placenta, we next examined whether ALK4 or ALK5 is involved in the GDF-11-induced downregulation of CGB expression [26]. To do this, a potent ALK4/5/7 inhibitor, SB431542, was used to block the function of ALK4 and ALK5 [27]. As shown in Fig. 2A, pretreatment of BeWo cells with SB431542 blocked the GDF-11-induced downregulation of CGB protein levels. Similarly, inhibition of ALK4 and ALK5 blocked the suppressive effect of GDF-11 on CGB protein in JEG-3 cells (Fig. 2B). To further determine which ALK is involved in GDF-11-induced downregulation of CGB expression, the siRNA-mediated knockdown approach was applied to eliminate the function of ALK4 or ALK5 specifically. As shown in Fig. 2C, RT-qPCR results revealed that transfection of BeWo cells with ALK4 siRNA specifically downregulated the endogenous ALK4 mRNA levels without affecting the endogenous ALK5 mRNA levels. The specificity of ALK5 siRNA was also observed. The suppressive effect of GDF-11 on CGB mRNA levels was partially attenuated by the knockdown of ALK4. Knockdown of ALK5 blocked the GDF-11-downregulated CGB mRNA levels. Western blot analysis confirmed the results that while both ALK4 and ALK5 were required for the GDF-11-induced downregulation of CGB protein levels, ALK5 was more involved in this process (Fig. 2D).

### GDF-11 downregulates CGB expression in primary human CTB cells

**Fig. 3:**
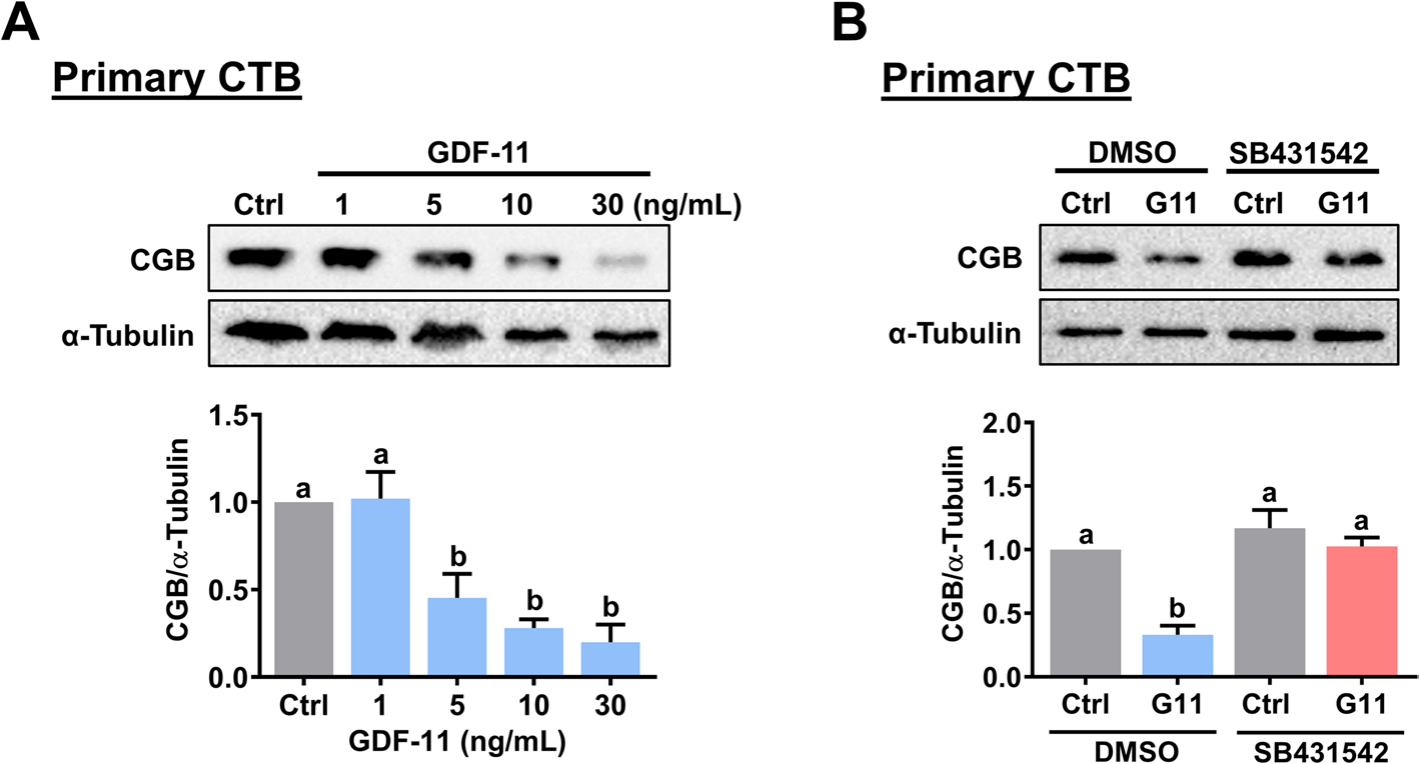
GDF-11 downregulates CGB expression in primary CTB cells. **A** Cells were treated with 1, 5, 10, and 30 ng/mL GDF-11 for 48 h. The protein levels of CGB were examined by western blot. **B** Cells were pretreated with vehicle control (DMSO) or 10 µM SB431542 for 1 h, and then treated with 10 ng/mL GDF-11 (G11) every 24 h for 48 h. The protein levels of CGB were examined by western blot. The results are expressed as the mean ± SEM of at least three independent experiments. The values without a common letter are significantly different (*p* < 0.05)

We are aware that choriocarcinoma cells may not fully represent the nature of human trophoblast cells. Therefore, the primary cytotrophoblast cells isolated from placental villi were used to further confirm the inhibitory effect of GDF-11 on CGB expression in human trophoblast cells. Under normal culture conditions, the primary CTB cells will differentiate into multinucleated syncytiotrophoblast cells and express CGB [28]. Similar to the results obtained from BeWo and JEG-3 cells, treatment with GDF-11 downregulated CGB protein levels in a concentration-dependent manner in primary CTB cells (Fig. 3A). In addition, the inhibitory effect of GDF-11 on CGB protein levels was blocked by the SB431542 (Fig. 3B).

### GDF-11 downregulates CGB expression by activating SMAD2/3 signaling pathways

**Fig. 4:**
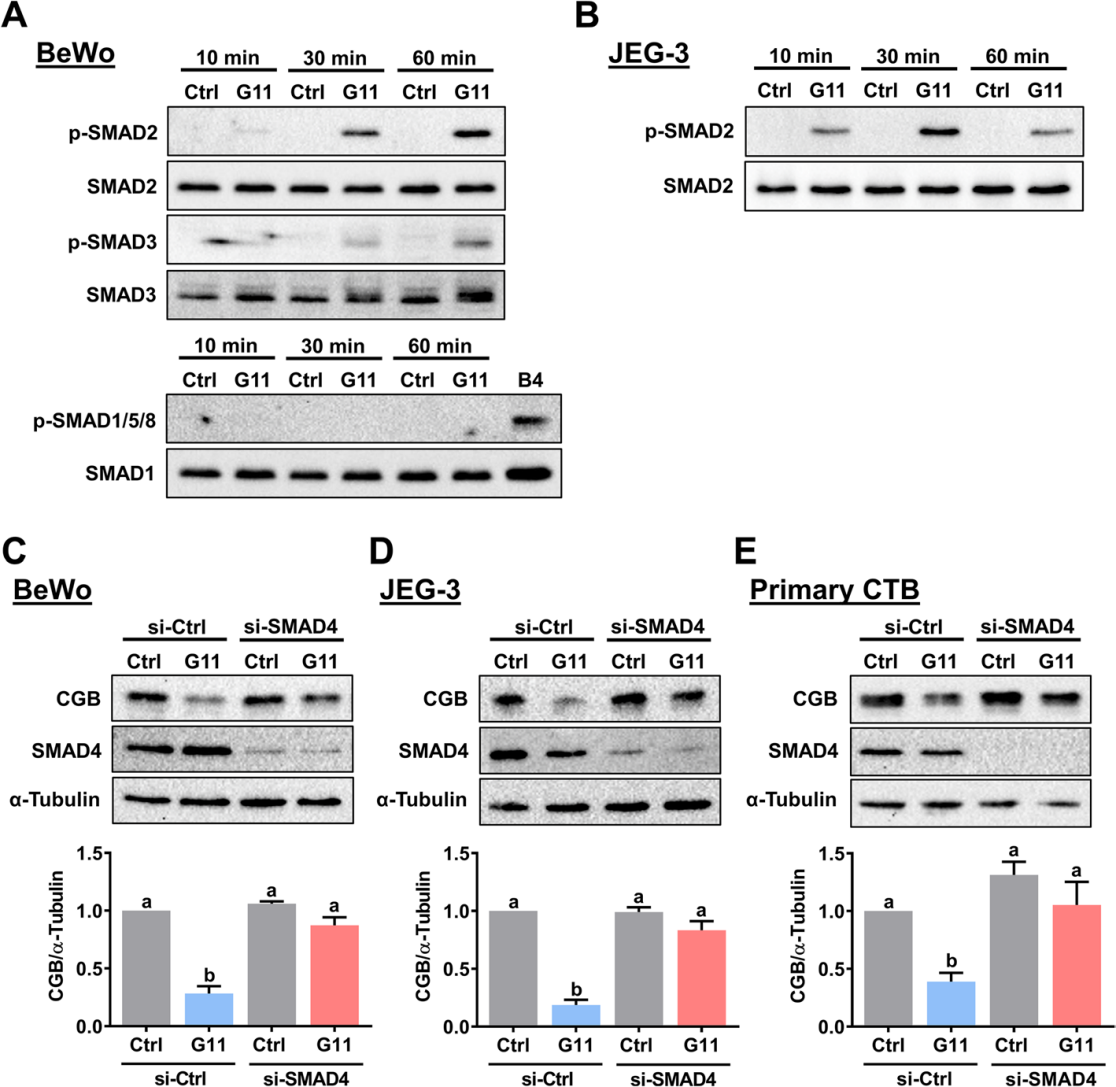
SMAD2 and SMAD3 mediate the GDF-11-induced downregulation of CGB expression. **A** BeWo cells were treated with 10 ng/mL GDF-11 (G11) for 10, 30, or 60 min. The levels of phosphorylated and total forms of SMAD2 and SMAD3 were determined by western blot (upper panel). The levels of phosphorylated forms of SMAD1/5/8 and the total form of SMAD1 were determined by western blot (lower panel). Cell lysate obtained from BeWo cells treated with 10 ng/mL BMP-4 (B4) for 60 min was used as a positive control. **B** JEG-3 cells were treated with 10 ng/mL GDF-11 (G11) for 10, 30, or 60 min. The levels of phosphorylated and total forms of SMAD2 were determined by western blot. **C-E** BeWo (**C**), JEG-3 (**D**), and primary CTB (**E**) cells were transfected with 50 nM (for BeWo and JEG-3) or 100 nM (for primary CTB) control siRNA (si-Ctrl) or SMAD4 siRNA (si-SMAD4) for 48 h, and then treated with 10 ng/mL GDF-11 (G11) every 24 h for 48 h. The protein levels of CGB and SMAD4 were examined by western blot. The results are expressed as the mean ± SEM of at least three independent experiments. The values without a common letter are significantly different (*p* < 0.05)

**Fig. 5:**
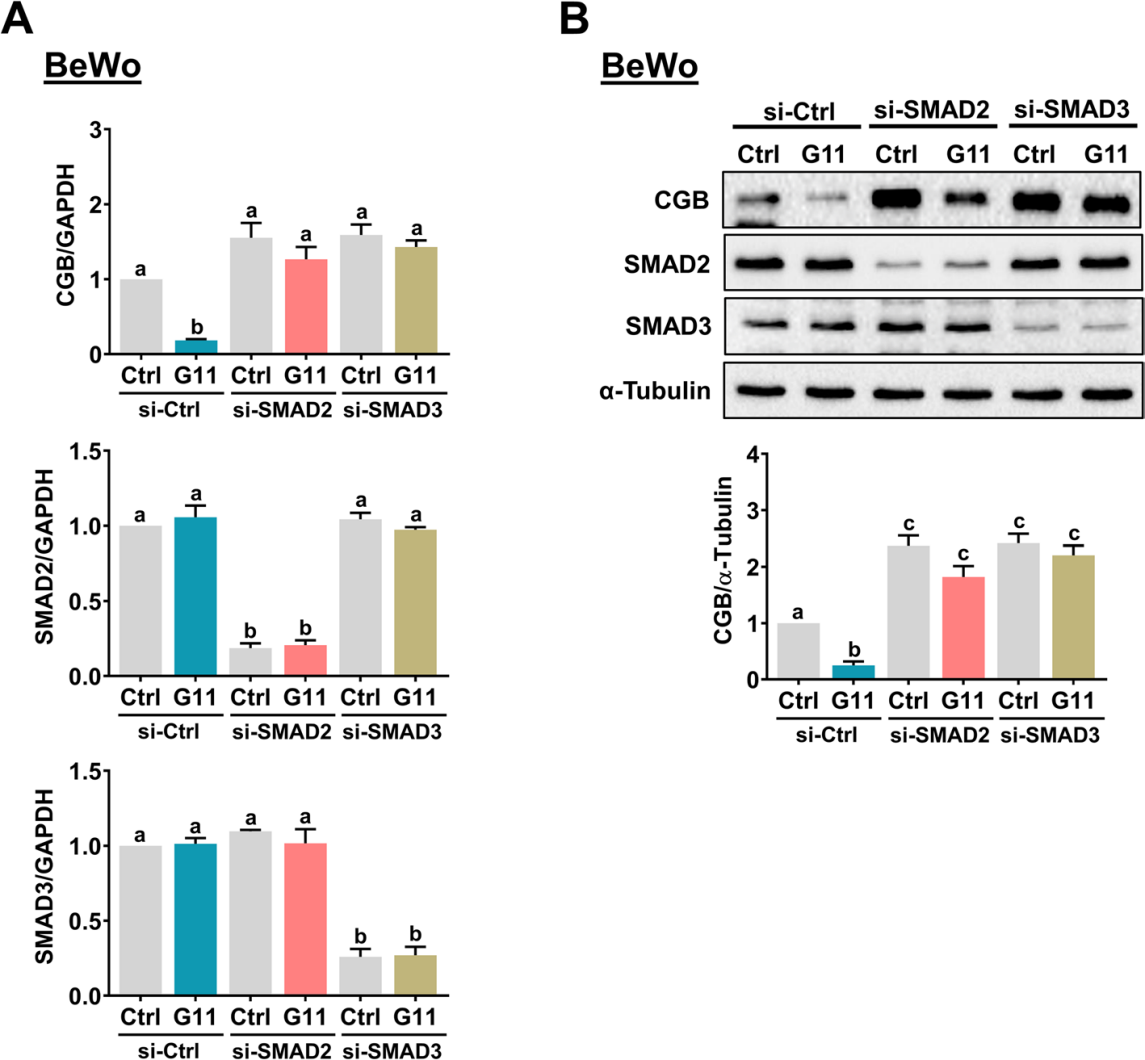
SMAD2 and SMAD3 are involved in the GDF-11-induced downregulation of CGB expression. **A** and **B** BeWo cells were transfected with 50 nM control siRNA (si-Ctrl), SMAD2 siRNA (si-SMAD2), or SMAD3 siRNA (si-SMAD3) for 48 h, and then treated with 10 ng/mL GDF-11 (G11) every 24 h for 48 h. The mRNA (**A**) and protein (**B**) levels of CGB, SMAD2, and SMAD3 were examined by RT-qPCR and western blot, respectively. The results are expressed as the mean ± SEM of at least three independent experiments. The values without a common letter are significantly different (*p* < 0.05)

Similar to other members of the TGF-β family, GDF-11 regulates cellular functions by activating intracellular SMAD2/3 or SMAD1/5/8 signaling pathways. Both SMAD2/3 and SMAD1/5/8 signaling pathways are activated in response to the GDF-11 treatment in human umbilical vein endothelial cells [29]. Therefore, we examined whether the same is true in BeWo cells. As shown in Fig. 4A, treatment with GDF-11 activated both SMAD2 and SMAD3 signaling pathways in BeWo cells. However, the activation of SMAD1/5/8 was not affected by the GDF-11 treatment. We used cell lysate obtained from BeWo cells treated with BMP-4 as a positive control for the activation of SMAD1/5/8 [21]. Loss of SMAD3 expression has been reported in JEG-3 cells [30]. Therefore, we confirmed the stimulatory effect of GDF-11 on SMAD2 in JEG-3 cells (Fig. 4B). As SMAD2/3 needs to form a complex with the common SMAD4 to regulate the expression of target genes, we knockdown the expression of SMAD4 to examine the requirement of SMAD2/3 in GDF-11-induced downregulation of CGB expression. As shown in Fig. 4C and D, knockdown of SMAD4 abolished the suppressive effect of GDF-11 on CGB protein levels in both BeWo and JEG-3 cells. Similarly, the inhibitory effect of GDF-11 on CGB protein levels in primary CTB cells was blocked by the knockdown of SMAD4 (Fig. 4E). Although in most contexts, the function of SMAD2 and SMAD3 are indistinguishable, these two signaling molecules can mediate distinct functions under some conditions [31]. Therefore, we next defined the individual role of SMAD2 and SMAD3 in mediating the suppressive effect of GDF-11 on CGB expression in BeWo cells. As shown in Fig. 5A, SMAD2 siRNA specifically downregulated the endogenous SMAD2 mRNA levels without affecting the endogenous SMAD3 mRNA levels and vice versa for SMAD3 siRNA. RT-qPCR results showed that the suppressive effect of GDF-11 on CGB mRNA levels was attenuated by the knockdown of SMAD2 or SMAD3. Western blot results confirmed the involvement of both SMAD2 and SMAD3 in the GDF-11-induced downregulation of CGB protein levels (Fig. 5B).

### GDF-11 decreases hCG production by activating ALK4/5-mediated SMAD2/3 signaling pathways

**Fig. 6:**
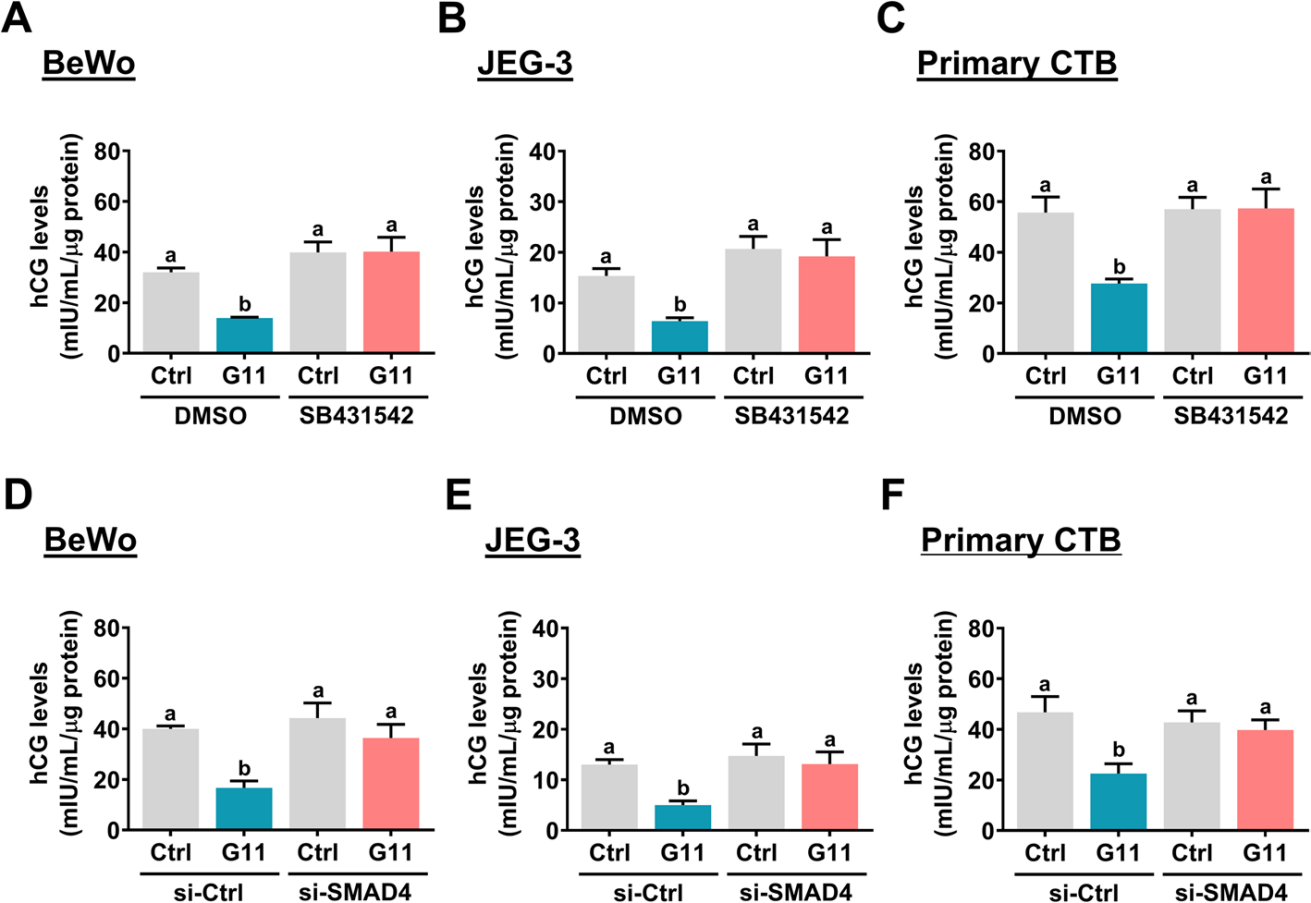
ALK4/5-mediated SMAD2/3 signaling pathways are required for the GDF-11-suppressed hCG production. **A-C** BeWo (**A**), JEG-3 (**B**), and primary CTB (**C**) cells were pretreated with vehicle control (DMSO) or 10 µM SB431542 for 1 h, and then treated with 10 ng/mL GDF-11 (G11) every 24 h for 72 h. **D-F,** BeWo (**D**), JEG-3 (**E**), and primary CTB (**F**) cells were transfected with 50 nM (for BeWo and JEG-3) or 100 nM (for primary CTB) control siRNA (si-Ctrl) or SMAD4 siRNA (si-SMAD4) for 48 h, and then treated with 10 ng/mL GDF-11 (G11) every 24 h for 72 h. The levels of hCG in the culture media were examined by ELISA. The results are expressed as the mean ± SEM of at least three independent experiments. The values without a common letter are significantly different (*p* < 0.05)

Given the suppressive effect of GDF-11 on CGB expression, we next examined whether the production of hCG is affected by GDF-11 treatment. ELISA was applied to measure the levels of hCG in the culture medium after the GDF-11 treatment. As shown in Fig. 6A and B, GDF-11 treatment significantly decreases the production of hCG in both BeWo and JEG-3 cells. In addition, the inhibitory effect of GDF-11 on hCG production was blocked by the inhibition of ALK4/5. SB431542 also abolished the inhibitory effect of GDF-11 on hCG production in primary CTB cells (Fig. 6C). Moreover, siRNA-mediated knockdown of SMAD4 also abolished the GDF-11-inhibited hCG production in both BeWo and JEG-3 cells as well as in primary CTB cells (Fig. 6D-F). Taken together, these results indicate that GDF-11 downregulates CGB expression by activating ALK4/5-mediated SMAD2/3 signaling pathways, which contributes to the decrease in hCG production.

## Discussion

There is no doubt that hCG plays a fundamental role in maintaining a normal pregnancy. The RNA of hCG can be detected as early as in the eight-cell stage of the embryo, and the hCG protein can be produced by blastocyst before implantation [32–34]. After implantation, although hCG can be secreted by extravillous trophoblast (EVT) cells, STB cells remain a major source of hCG [35]. Serum levels of hCG are rapidly increased and reach maximal levels at 8–10 weeks gestation. In addition to the well-known physiological functions required for pregnancy, excess hCG has been shown to be teratogenic to fetal gonadal tissues [36]. Therefore, the levels of hCG during pregnancy must be tightly regulated. It has been reported that the synthesis of hCG can be controlled by a few locally produced hormones and growth factors in an autocrine/paracrine manner [37]. The choriocarcinoma-derived cell lines, BeWo and JEG-3, are the commonly used cell models to study the synthesis of hCG. In the present study, using BeWo and JEG-3 cells, we demonstrated that the expression of CGB and production of hCG were inhibited by the GDF-11. In addition, the inhibitory effect of GDF-11 was further confirmed in human primary CTB cells. Mechanistically, we revealed that the suppressive effect of GDF-11 on CGB expression and hCG production was mediated by ALK4/5-SMAD2/3 signaling pathways.

Other groups and we have reported that EGFR ligands, EGF and AREG, can stimulate the expression of CGB expression and hCG production in human trophoblast cells [13, 38]. Many members of the TGF-β family are expressed in the human placenta and have important regulatory roles in placental development and disease [39]. Interestingly, different members of the TGF-β family exert a distinct effect on the CGB expression in different types of trophoblast cells. In the human immortalized EVT cell line, HTR-8/SVneo, the expression of CGB is upregulated in response to the TGF-β1 treatment [40]. In BeWo cells, the knockdown of transforming growth factor beta-induced factor-1 attenuated the expression of CGB and hCG secretion [41]. These results indicate the stimulatory role of TGF-β1 in hCG expression and secretion. In primary cultures of human placental cells, activin stimulates hCG secretion, while the secretion of hCG is inhibited by inhibin [42]. GDF-11 belongs to the GDF subfamily of the TGF-β superfamily. Our results showed that treatment with GDF-11 downregulated CGB expression and hCG production in both BeWo and JEG-3 cells. We were aware that BeWo and JEG-3 cells are derived from human choriocarcinoma, which can not fully represent the nature of normal human trophoblast cells. Therefore, in the present study, the inhibitory effect of GDF-11 on CGB expression and hCG production were further confirmed in primary human CTB cells.

We have shown that GDF-11 downregulates the expression of the steroidogenic acute regulatory protein in human granulosa cells by activating the ALK5-mediated SMAD3 signaling pathway [19]. Our recent study in human placental trophoblast cells reveals that GDF-11 stimulates cell invasion by upregulating matrix metalloproteinase-2 expression through ALK4/5-mediated SMAD2/3 signaling pathways [21]. These results indicate that the utilization of ALK4 or ALK5 receptor and SMAD2 or SMAD3 intracellular signaling by GDF-11 is in a cell type-dependent manner. In the present study, using a siRNA-mediated knockdown approach, our results demonstrated that both ALK4 and ALK5 were required for the GDF-11-suppressive CGB expression in human choriocarcinoma cells. In addition, SMAD2 and SMAD3 both were involved in the GDF-11-induced downregulation of CGB expression. As known as BMP-11, in addition to SMAD2/3, GDF-11 can activate SMAD1/5/8 signaling pathways [29]. We showed that BMP-4 treatment induced SMAD1/5/8 activation, which indicated these signaling pathways are not impaired in BeWo cells. However, we did not observe the stimulatory effect of GDF-11 on the activation of SMAD1/5/8 signaling pathways in BeWo cells. These results indicate that the cellular functions of GDF-11 in human choriocarcinoma cells are mediated by the SMAD2/3 but not SMAD1/5/8 signaling pathways. Similar to other members of the TGF-β superfamily, GDF-11 activates canonical SMAD signaling pathways and non-canonical signaling pathways such as ERK1/2, JNK, and p38 MPAK [43]. It has been shown that the expression of CGB in human trophoblast cells can be regulated by ERK1/2 and p38 MPAK [44]. Whether these signaling pathways can be activated by GDF-11 and mediate the GDF-11-induced downregulation of CGB expression in human trophoblast cells remain undetermined and warrant further investigation.

## Conclusions

In summary, in the present study, we provide evidence that the expression of CGB and production of hCG in human choriocarcinoma cells and primary CTB cells are downregulated in response to the treatment of GDF-11. Our results reveal that both ALK4 and ALK5 are required for the GDF-11-induced downregulation of CGB expression and hCG production. In addition, GDF-11 activates SMAD2/3 but not SMAD1/5/8 signaling pathways. Both SMAD2 and SMAD3 mediate the suppressive effects of GDF-11 on the expression of CGB and production of hCG. Our study not only discovers the biological function of GDF-11 in the human placenta but also provides important insights into the regulation of the hCG production.

## Data Availability

The data that support the findings of this study are available from the corresponding author upon reasonable request.

## Abbreviations

ALK: Activin receptor-like kinase
AREG: Amphiregulin
BMP: Bone morphogenetic protein
CTB: Cytotrophoblast cells
EGFR: Epidermal growth factor receptor
EVT: Extravillous trophoblast
GDF-11: Growth differentiation factor-11
hCG: Human chorionic gonadotropin
LH: Luteinizing hormone
STB: Syncytiotrophoblast cells
TGF-β: Transforming growth factor-β
